# Digital case-library and dual-mentor teaching for medical laboratory science internships in China: a historical comparison educational evaluation

**DOI:** 10.3389/fmed.2026.1876818

**Published:** 2026-07-06

**Authors:** Yawen Ding, Hong Zhang, Weixuan Zhang, Yan Wang, Huimin Zhang, Yipeng Wu, Ming Ma

**Affiliations:** 1 Department of Laboratory Medicine (East), The Fourth Hospital of Hebei Medical University, Shijiazhuang, Hebei, China; 2 College of Medical Technology, Hebei Medical University, Shijiazhuang, Hebei, China; 3 Department of Laboratory Medicine, The Fourth Hospital of Hebei Medical University, Shijiazhuang, Hebei, China

**Keywords:** case-based learning, China, clinical reasoning, competency-based education, dual mentoring, health professions education, internship, medical laboratory science education

## Abstract

**Background:**

Medical laboratory science internships often rely on fragmented rotation-based placements, limiting opportunities to connect laboratory results with clinical decision-making, quality-control reasoning and communication with clinicians. We evaluated the feasibility, acceptability and associated outcomes of a digital case-library-based dual-mentor teaching model.

**Methods:**

This single-centre retrospective historical controlled educational evaluation, not a randomised trial, included 36 interns from 2022 to 2023 who received routine rotation-based teaching and 30 interns from 2024 to 2025 who completed 10 structured digital case-library sessions. Clinical mentors introduced disease context and decision points; laboratory mentors guided test selection, result interpretation, quality-control reasoning and communication. Outcomes included archived historical records and questionnaires, intervention pre-post self-rated core competency, process indicators, blinded double-marked unseen-case objective assessment, mentor ratings and open-ended feedback.

**Results:**

Sixty-six interns were analysed. Sex distribution and grade point average strata did not differ significantly between groups. In the historical group, only 8.3% reported that routine rotations helped them link laboratory findings with clinical care, and no students reported enough case discussion or direct clinician communication. In the intervention group, 29 students had valid paired self-rating data; self-rated core-competency total increased from 14.62 ±3.18 to 38.55 ±4.19/55, with a mean gain of 23.93 points (95% CI 22.38–25.48; Wilcoxon W = 435.0, *p* < 0.001; *r* = 0.87). Attendance was 299/300 student-sessions. The blinded double-marked objective total averaged 85.50 ± 2.16/100, with good average-measure rater agreement [ICC(2, k) = 0.82, 95% CI 0.72–0.86]. End-of-placement self-rated core competency was higher in the intervention group than in the historical group (mean difference 2.86, 95% CI 0.50–5.21; *p* = 0.007; Hedges g = 0.58, 95% CI 0.14–1.10), whereas routine exit marks did not differ significantly.

**Conclusion:**

The model was feasible and well accepted. Findings suggest an associative trend in perceived competency development with preliminary convergent evidence, but causal interpretation is limited by retrospective approval, non-random historical comparison, absent historical baseline measurements and reliance on self-rated outcomes.

## Introduction

Competency-based education in medical laboratory science requires more than mastery of analytical methods and routine bench work. During internships, students are expected to progress from performing tests to explaining abnormal findings, supporting clinical decision-making, recognising analytical and quality-control risks, and communicating effectively with clinicians. Biomedical science and health-professions education research has highlighted a persistent theory-practice gap in helping students understand how laboratory work contributes to patient care, multidisciplinary decision-making and professional practice (1, 2). Similar concerns have also been noted in mainland China, where training frameworks increasingly emphasise competency-oriented reform but much internship teaching remains dominated by departmental rotation and knowledge transmission (3, 4).

Case-based and problem-based pedagogies offer a plausible response to this gap. In biomedical science, medical laboratory science and allied health education, case study presentations, problem-based learning and active learning have been associated with improved engagement, deeper understanding and stronger application of disciplinary knowledge to authentic practice scenarios (4–8). These approaches are also compatible with workplace learning, because they can situate laboratory interpretation within patient stories, decision points, feedback and communication tasks.

However, the specific implementation gap in mainland China medical laboratory science internships remains under-described. Existing domestic teaching reforms have most often used problem-based or case-based learning within specific clinical laboratory courses, or section-specific supervision, and many workplace placements still rely on a single mentor or informal bench-level guidance. Few published models have been tailored to the small-cohort feature of Chinese medical laboratory internships by integrating a reusable digital case library with explicit division of clinical and laboratory mentor roles and aligned case-linked assessment.

For these placements, educational innovation faces several practical constraints. Annual cohorts at a single centre are usually small, students rotate through multiple subspecialty benches, and opportunities for repeated cross-disciplinary discussion are limited. Traditional placements may therefore support operational familiarity without reliably developing the linked skills of test selection, result interpretation, discrepancy investigation and laboratory-clinician communication (3, 4, 9).

To address this specific gap, we developed a digital case library and implemented a dual-mentor teaching model in which a clinical mentor introduced the disease context and decision points while a laboratory mentor guided test selection, interpretation, quality-control reasoning and communication. We hypothesised that this structured model would be feasible in a small-cohort internship setting and would be associated with more favourable self-rated laboratory-clinical reasoning and preliminary case-linked performance indicators than routine rotation-based teaching.

## Materials and methods

### Study design and setting

This was a single-centre retrospective historical controlled educational evaluation with open-ended learner feedback, undertaken at The Fourth Hospital of Hebei Medical University, Shijiazhuang, China. It was not a randomised controlled trial. Quantitative analyses combined a historical comparison between routine rotation-based teaching and dual-mentor teaching conditions with a within-group pre-post evaluation in the intervention cohorts. Open-ended comments were coded into high-frequency perceived strengths and areas for improvement.

### Participants and sample size

The sample was pragmatic and included all eligible interns available within four consecutive cohorts at one centre. The historical comparison group comprised 36 interns from the 2022 and 2023 cohorts (20 and 16 students, respectively) who had completed traditional rotation-based placements. All 36 eligible students had archived end-of-placement assessment records and end-of-placement questionnaires available for analysis. These questionnaires were administered contemporaneously at the end of the laboratory medicine placement or relevant laboratory training block on 17 February 2023 and 28 February 2024 and were analysed retrospectively within the present study. The intervention group comprised 30 interns from the 2024 and 2025 cohorts (15 in each cohort) who received the digital case-library-based dual-mentor programme. Inclusion required availability of the relevant questionnaire and/or routine end-of-placement scores. No formal a priori sample size calculation was undertaken because annual intake in this specialty is small and centre-level educational randomisation was not feasible; we therefore used an all-available-cohort approach.

### Teaching conditions

The historical comparison group underwent routine rotation-based teaching across laboratory sections such as haematology, biochemistry, immunology, microbiology and transfusion. Teaching mainly consisted of section-specific demonstrations, knowledge explanation and day-to-day bench supervision. There was no standardised digital case library, no fixed clinical mentor and no cross-section case-discussion structure. The intervention group received 10 structured sessions based on the first 10 cases in a self-developed digital case library. Topics covered iron deficiency versus thalassaemia, acute leukaemia, blood-gas interpretation in chronic obstructive pulmonary disease, diabetic ketoacidosis, EDTA-dependent pseudothrombocytopenia, coagulation abnormalities, jaundice patterns, false versus true creatinine elevation, discordance between HbA1c and red-cell lifespan, and causes of hypoglycaemia. Each session followed the sequence digital pre-reading, guided question chain, small-group discussion, student reporting, dual-mentor feedback and post-session consolidation. The clinical mentor introduced the patient story and clinical decision context, whereas the laboratory mentor focused on first-line test selection, interpretation of abnormal results, analytical and quality-control reasoning, and laboratory-clinician communication. For the 2025 cohort, the post-programme self-rated core-competency assessment and the post-course objective assessment were completed on 21 January 2026 before the graduation examination to minimise potential examination-related influence on post-course performance.

### Outcome measures and data sources

For the historical comparison group, data comprised archived routine exit scores, an end-of-placement 11-item self-rated core-competency questionnaire scored from 1 to 5, an 8-item binary questionnaire on traditional teaching experience and an additional non-parallel case test retained for descriptive reporting only. The historical cohorts did not have baseline measurements using the same 11-item self-rated instrument. Therefore, change scores and difference-in-differences analyses could not be performed. For the intervention group, final verified data comprised the 11-item pre-post self-rated core-competency total score, ten-session process indicators, a post-course unseen-case objective assessment scored independently by two blinded raters, course acceptance count, mentor overall/communication/quality-control ratings and open-ended comments. The analytic role and inferential boundary of each outcome are shown in Table 1. The assessment instruments, scoring anchors, unseen-case rubric, content-validity documentation, de-identified datasets and analysis reproducibility files are provided in the Supplementary material.

**TABLE 1:** Analytic role of outcomes and inference boundaries.

Outcome/data source	Primary use in this study	Analytic role and inference boundary
Sex and grade point average strata	Historical and intervention groups	Baseline comparability only; non-significant differences do not prove balance.
11-Item self-rated core competency	Intervention pre-post analysis and end-of-placement historical comparison	Formal self-rated/perceived competency outcome; no historical baseline, so no change-score or difference-in-differences comparison.
Routine exit marks	End-of-placement historical comparison	Formal descriptive/inferential comparison of routine assessment marks; possible ceiling effect and limited sensitivity.
Historical teaching-experience questionnaire	Historical group only	Contextual description of gaps in routine teaching.
Process indicators and course acceptance	Intervention group only	Feasibility, engagement and acceptability indicators; not independent evidence of learning effectiveness.
Unseen-case objective assessment	Intervention group only	Post-course case-linked performance and rater reliability; no equivalent historical comparison.
Mentor ratings and correlations	Intervention group only	Preliminary convergent evidence with self-rated and objective indicators.
Historical non-parallel case test	Historical group only	Descriptive only; not comparable with the intervention objective assessment.

### Instrument, rubric and feedback procedures

The questionnaire and unseen-case rubric are described as locally developed, content-validity-supported instruments/rubrics. They were developed from the intended competency domains of the intervention and used for educational evaluation rather than as externally validated instruments. A seven-expert panel comprising medical laboratory educators, clinical laboratory specialists, clinical mentors and educational-management staff reviewed relevance and clarity. Content-validity index (CVI) values of 1.00 refer to the final versions after wording standardisation and panel feedback. The unseen-case rubric included information extraction, understanding of the laboratory question, first-round test selection, result interpretation, authenticity/quality-control judgement, next investigative step, support for clinical decision-making and communication. Two raters independently scored anonymised post-course scripts. Raters were blinded to student identity, cohort/year, self-rated scores, mentor ratings and routine exit marks. They received the written rubric and scoring anchors before scoring; no formal calibration dataset or domain-level rater file was retained in the final verified dataset, so reliability is reported for total scores only. The final objective score was calculated directly as the mean of the two raters’ total scores. Item-level data were retained for the historical end-of-placement self-rated questionnaire, for which Cronbach’s alpha was 0.92; this statistic was used to describe internal consistency and was not interpreted as evidence of external validity (10). The final verified intervention dataset retained pre- and post-course total scores rather than item-level self-rating scores, so intervention alpha values were not recalculated.

Open-ended responses were analysed as pragmatic educational feedback rather than as a full qualitative interview study, with the limited goal of identifying recurring information-rich themes from de-identified course comments (11). Two researchers independently reviewed the de-identified response summaries, coded recurring content into high-frequency themes, compared coding, and resolved discrepancies by discussion with a third author. Final themes were generated by combining similar codes and counting the number of students whose comments matched each theme. Raw free-text quotations were not included in the submitted individual-level datasets to reduce re-identification risk; therefore, results are reported as coded high-frequency themes rather than verbatim quotations.

### Statistical analysis

Data were cleaned and analysed in Python 3.13. Categorical variables are presented as counts and percentages, and continuous variables as mean ± standard deviation with 95% confidence intervals (CIs) for key estimates where appropriate. Sex was compared using the chi-square test with Yates correction, and grade point average strata were compared using Pearson chi-square testing. Within the intervention group, paired self-rated core-competency total scores were compared with the Wilcoxon signed-rank test, and an effect size (*r* = Z/sqrt[N]) was calculated for the total-score change. Complete-case analysis was used for paired self-rating because one student did not have a verified post-course self-rating total in the final submission dataset; that student’s objective, mentor-rated and routine placement outcomes were retained for analyses not involving self-rated competency. Because the historical comparison group lacked baseline self-rated measurements, the study could not compare change scores or perform a difference-in-differences analysis. End-of-placement comparisons between the historical and intervention groups used the Mann-Whitney U test. For the principal between-group end-of-placement self-rated core-competency comparison, we report mean difference, 95% CI and Hedges g. As a sensitivity analysis, the end-of-placement self-rated core-competency comparison was additionally modelled using ordinary least-squares regression adjusted for sex and grade point average stratum with heteroscedasticity-robust HC3 standard errors. Agreement between two blinded raters for the post-course objective total was examined using a two-way random-effects intraclass correlation coefficient for absolute agreement (single- and average-measure ICC). Spearman rank correlations were used within the intervention cohort to explore convergent associations between self-rated, objective, mentor-rated and routine placement outcomes. Bootstrap 95% CIs were calculated for Hedges g, ICCs and Spearman correlations. Two-sided *p*-values < 0.05 were considered statistically significant.

### Ethical considerations

The retrospective analysis and reporting of this educational-evaluation dataset were approved by the Ethics Committee of The Fourth Hospital of Hebei Medical University on 21 April 2026 (approval No. 2026KS078) within the project “Strategies for Training Medical Laboratory Students Through a Dual-Mentor Model Based on Integrated Teaching.” The approval was obtained after completion of the teaching activities and before manuscript submission, and the present report is therefore described as a retrospectively approved educational evaluation rather than a prospectively approved trial. The Ethics Committee explicitly approved the retrospective use and reporting of coded, de-identified educational records, historical questionnaires and course-feedback data, and waived the requirement for additional written informed consent for this retrospective analysis. Questionnaire/course-feedback participation was voluntary, the study was conducted in accordance with local legislation and institutional requirements, and no identifiable student or patient data are reported.

## Results

Sixty-six interns were analysed: 36 in the historical comparison group and 30 in the dual-mentor intervention group. Sex distribution and grade point average strata did not differ significantly between groups (Table 2). In the intervention group, 29 students attended all 10 sessions and one missed one session, giving an overall attendance rate of 299/300 student-sessions (99.7%). Valid paired self-rating data were available for 29 intervention students because one post-course self-rating total was not retained in the final verified dataset.

**TABLE 2:** Participant characteristics and group comparability.

Characteristic	Historical comparison group (*n* = 36)	Dual-mentor intervention group (*n* = 30)	*P*-value
Analysed students	36	30	−
Cohorts	2022: 20; 2023: 16	2024: 15; 2025: 15	−
Female	19 (52.8%)	18 (60.0%)	0.734
Male	17 (47.2%)	12 (40.0%)	0.734
Grade point average stratum A	11 (30.6%)	6 (20.0%)	0.437
Grade point average stratum B	16 (44.4%)	18 (60.0%)	0.437
Grade point average stratum C	9 (25.0%)	6 (20.0%)	0.437

Traditional rotation-based teaching was perceived as weak in helping students integrate laboratory findings with clinical care. Only 36.1% of historical respondents felt that learning objectives were clear, 8.3% thought that content was broadly consistent across rotations, and 8.3% felt that traditional teaching helped them understand the laboratory-clinical link. No respondent reported sufficient opportunities for case discussion or direct communication with clinicians. Nevertheless, 77.8% said they would have chosen a case-library-plus-dual-mentor model had it been available (Table 3). Open-ended comments highlighted the need for structured discussion of abnormal results, discrepant findings, pre-analytical problems and simulated clinician communication.

**TABLE 3:** Historical group appraisal of routine rotation-based teaching.

Item	Result
Learning objectives during rotations were clear	13/36 (36.1%)
Teaching content was broadly consistent across sections	3/36 (8.3%)
Traditional teaching helped link laboratory findings with clinical care	3/36 (8.3%)
Feedback was timely and specific	8/36 (22.2%)
There were enough opportunities for case discussion	0/36 (0.0%)
There were enough opportunities for direct communication with clinicians	0/36 (0.0%)
Overall task load was acceptable	5/36 (13.9%)
Would have chosen digital case-library teaching with dual mentors if available	28/36 (77.8%)
Traditional teaching support count (0–8)	1.67 ± 0.99

Before the intervention, all 30 students had no prior exposure to joint clinical-laboratory teaching. Most had only a vague understanding that laboratory results should be interpreted together with symptoms and clinical context, and two-thirds reported little or no confidence in telephoning clinicians about suspicious results. Across the 10-session programme, engagement remained high, with 299 of 300 planned student-sessions completed. Mean formative score was 23.22 ± 0.30/30, platform completion was 98.28 ± 0.19%, and interactive-question accuracy was 98.31 ± 0.17% (Table 4). These process indicators are interpreted as feasibility and learner-engagement measures rather than independent evidence of learning effectiveness.

**TABLE 4:** Intervention pre-course status and process indicators.

Indicator	Result
No previous exposure to joint clinical-laboratory teaching	30/30 (100.0%)
Only a vague understanding that laboratory results should be interpreted with symptoms	24/30 (80.0%)
No understanding of this principle	6/30 (20.0%)
Would immediately repeat a result assuming personal operational error	25/30 (83.3%)
Would review specimen/clinical interference and contact clinicians	1/30 (3.3%)
Little or no confidence in calling clinicians about suspicious results	20/30 (66.7%)
Attendance across 10 sessions	299/300 student-sessions (99.7%)
Formative assessment score (0–30)	23.22 ± 0.30
Platform completion (%)	98.28 ± 0.19
Interactive-question accuracy (%)	98.31 ± 0.17

Among 29 students with valid paired self-rating totals, the 11-item self-rated core-competency total score increased from 14.62 ± 3.18 to 38.55 ± 4.19/55, a mean gain of 23.93 points (95% CI 22.38–25.48; Wilcoxon *W* = 435.0, *p* < 0.001; effect size *r* = 0.87; Table 5). Because historical cohorts did not have baseline self-rated core-competency measurements, this finding represents within-intervention change in perceived competency and cannot be used to compare change over time between groups.

**TABLE 5:** Self-rated core-competency total score before and after the programme.

Outcome	Pre-course	Post-course	Mean difference (95% CI)	Test	Statistic	*P*-value
Self-rated core-competency total score (11 items; range 11–55; valid pairs *n* = 29)	14.62 ± 3.18	38.55 ± 4.19	23.93 (22.38 –25.48)	Wilcoxon signed-rank	W = 435.0	< 0.001

Compared with the historical comparison group, the intervention group had a higher end-of-placement self-rated core-competency score (38.55 ± 4.19 vs. 35.69 ± 5.31/55; mean difference 2.86, 95% CI 0.50–5.21; Mann-Whitney *U* = 318.0, *p* = 0.007; Hedges *g* = 0.58, bootstrap 95% CI 0.14–1.10). This result was similar in the covariate-adjusted sensitivity analysis: after adjustment for sex and grade point average stratum, intervention-group membership remained associated with a higher end-of-placement self-rated core-competency score (adjusted coefficient 2.87, robust 95% CI 0.66–5.08; *p* = 0.011). Routine exit marks were high in both groups and did not differ significantly for theory, practical/skills performance, case discussion/presentation, daily performance or overall exit score (Table 6). Historical supplementary case tests yielded a mean score of 71.56 ± 2.21/100 (95% CI 70.81–72.30), whereas the intervention group achieved 85.50 ± 2.16/100 (95% CI 84.69–86.31) on the post-course objective assessment; this contrast is descriptive only and not comparable with the intervention objective assessment because the historical case versions were not equivalent or parallel.

**TABLE 6:** End-of-placement group comparisons and sensitivity analysis.

Outcome	Historical comparison group	Dual-mentor intervention group	Estimate	Test	Statistic	*P*-value
End-of-placement self-rated core-competency score (11–55; intervention valid *n* = 29)	35.69 ± 5.31	38.55 ± 4.19	Mean difference 2.86 (95% CI 0.50– 5.21); Hedges g 0.58 (95% CI 0.14 to 1.10)	Mann-Whitney U	318.0	0.007
Adjusted sensitivity analysis for self-rated core competency	−	−	Adjusted coefficient 2.87 (95% CI 0.66– 5.08)	OLS with HC3 SE	−	0.011
Exit theory mark (0–100)	90.22 ± 4.08	90.27 ± 2.13	−	Mann-Whitney U	553.0	0.871
Practical or skills mark (0–100)	89.78 ± 4.01	90.90 ± 2.48	−	Mann-Whitney U	434.5	0.174
Case discussion or presentation mark (0–100)	90.58 ± 4.08	89.93 ± 2.59	−	Mann-Whitney U	610.5	0.365
Daily performance mark (0–100)	90.44 ± 3.82	91.43 ± 2.54	−	Mann-Whitney U	476.0	0.411
Overall exit mark (0–100)	90.03 ± 4.13	91.07 ± 1.96	−	Mann-Whitney U	477.5	0.422

Historical supplementary case-test results are descriptive only and not comparable with the intervention objective assessment because the case versions were not equivalent or parallel.

Acceptability in the intervention group was high. The mean course-acceptance count was 7.67 ± 0.61/8. The blinded double-marked post-course objective total averaged 85.50 ± 2.16/100. Agreement was moderate for a single rater and good for the average of two raters (ICC[2,1] = 0.69, 95% CI 0.57–0.76; ICC[2,k] = 0.82, 95% CI 0.72–0.86). Mentor ratings were above 4/5 for overall performance, communication and quality-control reasoning. Open-ended responses most often praised discussion of what to do when laboratory results did not fit the clinical picture and clinically oriented interpretation of abnormal results. The most common suggestions for improvement were to reduce the number of process-assessment items, moderate the clinical complexity of some cases and make communication training more realistic (Table 7).

**TABLE 7:** Intervention acceptability, objective scoring, mentor ratings, correlations and open-ended themes.

Item	Result
Course-acceptance count (0–8)	7.67 ± 0.61
Unseen post-course objective score (0–100)	85.50 ± 2.16 (95% CI 84.69–86.31)
Single-rater agreement	ICC[2, 1] = 0.69 (95% CI 0.57–0.76)
Average-measure rater agreement	ICC[2, k] = 0.82 (95% CI 0.72–0.86)
Mentor rating: overall mean (0–5)	4.23 ± 0.17
Mentor rating: communication mean (0–5)	4.15 ± 0.35
Mentor rating: quality-control reasoning mean (0–5)	4.23 ± 0.34
Post self-rated competency vs. objective total	Spearman rho = 0.47 (95% CI 0.11–0.75), *p* = 0.011; *n* = 29
Post self-rated competency vs. mentor overall	Spearman rho = 0.54 (95% CI 0.17–0.83), *p* = 0.002; *n* = 29
Objective total vs. mentor overall	Spearman rho = 0.56 (95% CI 0.25–0.80), *p* = 0.001; *n* = 30
Objective total vs. overall exit mark	Spearman rho = 0.77 (95% CI 0.59–0.89), *p* < 0.001; *n* = 30
Most helpful theme: discussing results that do not fit the clinical picture	15/30 (50.0%)
Most helpful theme: clinically oriented interpretation of abnormal results	10/30 (33.3%)
Main suggestion: too many process-assessment items	10/30 (33.3%)
Main suggestion: some cases were clinically too complex	9/30 (30.0%)
Main suggestion: communication training could be more realistic	7/30 (23.3%)

Within the intervention cohort, higher post-course self-rated competency correlated with higher objective totals (Spearman rho = 0.47, 95% CI 0.11–0.75; *p* = 0.011; *n* = 29) and higher mentor overall ratings (rho = 0.54, 95% CI 0.17–0.83; *p* = 0.002; *n* = 29). Objective totals also correlated with mentor overall ratings (rho = 0.56, 95% CI 0.25–0.80; *p* = 0.001; *n* = 30) and overall exit marks (rho = 0.77, 95% CI 0.59–0.89; *p* < 0.001; *n* = 30), providing preliminary convergent evidence that the self-rated gains were not entirely independent of external performance indicators.

## Discussion

This study extends a dual-mentor educational framework into a pragmatic internship intervention and shows that it can be implemented in a small-cohort mainland China setting with high engagement and strong learner acceptance. The historical comparison data also add an important contextual dimension: traditional rotation-based placements were not perceived to provide enough structured case discussion, laboratory-clinical integration or direct clinician communication, whereas the dual-mentor case-based model directly targeted these gaps.

The study should be interpreted as a retrospective historical controlled educational evaluation, not as a randomised controlled trial. Accordingly, the results indicate an associative trend between the teaching model and more favourable perceived laboratory-clinical reasoning outcomes, rather than definitive causal superiority over routine rotation-based teaching. This distinction is particularly important because the historical cohorts lacked baseline self-rated measurements and an equivalent objective assessment.

The intervention group showed a large within-group increase in self-rated core competency. This pattern is consistent with biomedical science and health-professions education literature identifying the need for teaching that links laboratory work more explicitly to patient outcomes, multidisciplinary practice and authentic workplace reasoning (1, 2, 5, 6). In our setting, pairing a clinical mentor with a laboratory mentor appeared to support reasoning about discrepant results, quality-control risk and explanations relevant to downstream clinical decisions. However, this evidence remains perception-based and should be understood as development in self-rated or perceived competency, not direct proof of independently measured competence.

At the same time, the strongest signal in the present study still came from a self-rated instrument. Self-rated competency should not be equated with independently measured competency, because expectancy effects, Hawthorne effects and response-shift bias may all have contributed to the magnitude of the observed pre-post change. The post-course objective total was derived from two blinded raters with good average-measure agreement, higher self-rated scores were moderately associated with both objective totals and mentor ratings, and the locally developed instruments had expert-panel content-validity support. The findings are therefore best interpreted as perceived competency development with preliminary convergent and content-validity support, rather than definitive proof of superiority on fully standardised external performance measures.

The comparison between historical and intervention cohorts was more informative when outcomes reflected laboratory-clinical reasoning than when they used routine placement marks. End-of-placement self-rated core-competency scores were higher in the intervention group than in the historical comparison group, and the sensitivity analysis adjusted for sex and grade point average stratum showed a similar association. Nevertheless, unmeasured temporal and cohort differences remain possible. Routine exit marks were high in both groups and did not differ significantly. This likely reflects limited sensitivity of routine placement marks, which cluster at high levels and prioritise completion of everyday bench tasks over the laboratory-clinical reasoning, quality-control reasoning and clinician-communication competencies targeted by the intervention. This interpretation also aligns with biomedical science education studies showing that active learning, assessment literacy, placement support and responsible assessment design in an AI era can influence engagement, performance, graduate attributes and workplace readiness (12–15). Reviews of clinical reasoning curricula, case-based learning and clinical reasoning assessment in health-professions education further suggest that repeated exposure to authentic cases, feedback and construct-aligned assessment are central mechanisms for strengthening applied reasoning in workplace-oriented training (16–18).

This work also has methodological relevance for health-professions education programmes with small annual cohorts. Medical laboratory science placements in mainland China often make large randomised educational trials difficult. Our design combined four consecutive cohorts, a historical comparison group, repeated measures within the intervention cohorts, process learning data and open-ended feedback. While not eliminating bias, this design produced interpretable educational-evaluation evidence within the constraints of a real training environment and may be useful for other small-cohort programmes seeking to evaluate teaching change. The wider biomedical science education literature also supports digitally enabled scenario-based learning and structured mentoring/pipeline support as ways to strengthen student engagement and workforce development (19, 20).

## Limitations

The study has several limitations. First, it was single-centre, non-randomised and historically controlled, so time trends, faculty changes, teacher learning effects, internship-policy adjustments, secular changes in placement conditions and unmeasured cohort differences cannot be excluded. The results therefore support only associative educational-evaluation evidence, not causal effectiveness. Second, the historical comparison group had only end-of-placement self-rated core-competency data and lacked baseline measurements using the same instrument; change-score comparison and difference-in-differences analysis were therefore impossible. The intervention pre-post finding should be interpreted as within-intervention improvement in perceived competency, not as evidence that the magnitude of change exceeded natural development under routine rotation-based teaching. Third, ethics approval for retrospective analysis and reporting was obtained after completion of the teaching activities; however, the Ethics Committee explicitly approved the retrospective use and reporting of coded, de-identified educational-evaluation data and waived the requirement for additional written informed consent. This design should therefore be interpreted as a retrospectively approved educational evaluation rather than as a prospectively approved trial. Fourth, although the historical-group questionnaires were completed contemporaneously at the end of the laboratory medicine placement or relevant training block rather than re-administered years later, the historical comparison design remains vulnerable to bias from between-cohort differences and changes in the teaching environment over time. Fifth, the historical supplementary case test was not a true parallel paper to the intervention objective assessment and was retained for descriptive reporting only; it was not used for formal objective between-group inference. Sixth, the strongest gain was demonstrated in a self-rated competency instrument rather than in fully standardised external performance measures. One intervention student did not have a verified post-course self-rating total in the final submission dataset, reducing the paired self-rating sample to 29 students and illustrating the sensitivity of questionnaire-based outcomes to source-data quality. Seventh, the core-competency questionnaire and unseen-case rubric were locally developed, content-validity-supported tools. The historical item-level questionnaire showed high internal consistency, and the objective total showed good average-measure inter-rater agreement, but broader external validity evidence, intervention item-level reliability and domain-level objective reliability were not available. Finally, the pragmatic sample size was small and no a priori sample size calculation was performed; the study may therefore have been underpowered for small-to-moderate between-group effects, particularly for conventional exit marks that showed ceiling effects.

## Conclusion

A digital case-library and dual clinical-laboratory mentor model was feasible and well accepted in a small-cohort medical laboratory science internship programme in China. The model was associated with higher perceived laboratory-clinical reasoning and supported by preliminary convergent evidence from blinded objective scoring and mentor ratings. These findings do not establish causal superiority over routine rotation-based teaching but support further prospective, multi-centre evaluation using baseline measurements, parallel objective assessments, standardised external performance measures and stronger designs for causal inference.

## Data Availability

The original contributions presented in the study are included in the article/Supplementary material, further inquiries can be directed to the corresponding author.

## Supplementary material

Supplementary File 1 De-identified datasets and Supplementary Tables S1–S5, including the dual-rated unseen-case objective assessment, verified intervention master dataset, historical data-source and bias audit, and historical individual-level dataset.

Supplementary File 2 Assessment instruments, scoring anchors, unseen-case final-assessment instructions and 100-point rubric, blinded scoring procedure, and content-validity documentation.

Supplementary File 3 Updated analysis reproducibility package including the Python script, README, requirements, de-identified input datasets, output log and SHA256 manifest; the package reproduces the confidence intervals and covariate-adjusted sensitivity analysis.
