# Supplementary material for: Digital case-library and dual-mentor teaching for medical laboratory science internships in China: a historical comparison educational evaluation
Source: Front Med (Lausanne). 2026 Jul 6;13:1876818. doi: 10.3389/fmed.2026.1876818 (PMC13382507; doi:10.3389/fmed.2026.1876818)
Supplement: Supplementary File 2 — Assessment instruments, scoring anchors, unseen-case final-assessment instructions and 100-point rubric, blinded scoring procedure, and content-validity documentation. [file Data_Sheet_2.pdf]

# Supplementary File 2

## Instruments, scoring anchors, unseen-case final-assessment rubric and content-validity documentation

Manuscript: Digital Case-Library and Dual-Mentor Teaching for Medical Laboratory Science Internships in China: A Historical Comparison Educational Evaluation.

Purpose of this file: This revised supplementary file provides the assessment tools and scoring documentation requested during editorial checking. It includes the student questionnaires, scoring anchors, mentor rating anchors, the final unseen-case assessment instructions and answer prompts, the 100-point scoring rubric used for the final objective assessment, the blinded scoring procedure and content-validity documentation. The instruments are translated into English for peer review and publication purposes. Student identifiers and patient-identifiable details are not included.

## S2.1 Overview of instruments and assessment materials

| Instrument or material | Respondent / rater | Timing | Scoring and analytic role |
|---|---|---|---|
| 11-item self-rated core-competency questionnaire | Student interns | Historical cohorts: end of laboratory medicine placement/relevant training block; intervention cohorts: before and after the programme | Each item scored 1-5; total 11-55. Used as self-rated/perceived competency outcome. |
| Historical teaching-experience questionnaire | Historical-group student interns | End of laboratory medicine placement/relevant training block | Eight yes/no items; support count 0-8. Used to describe perceived gaps in routine teaching. |
| Intervention course-acceptability questionnaire | Intervention-group student interns | After the 10-session programme | Eight yes/no items; acceptance count 0-8. Used for acceptability and engagement. |
| Mentor rating form | Clinical/laboratory mentors | End of programme | Ratings from 1 to 5. Final verified dataset retained overall, communication and quality-control reasoning means. |
| Final unseen-case objective assessment | Intervention-group student interns | After teaching sessions and before graduation examination for the 2025 cohort | Written case-based assessment. Two blinded raters independently scored anonymised scripts; final score = mean of two total scores. |
| Open-ended feedback prompts | Intervention-group student interns | After the programme | Free-text responses coded as high-frequency themes; raw quotations withheld to reduce re-identification risk. |

## S2.2 Scoring anchors for the 11-item self-rated core-competency questionnaire

| Score | Anchor | Operational meaning |
|---|---|---|
| 1 | Not yet able | Unable to perform the competency independently; requires complete guidance. |
| 2 | Initial understanding | Recognises the topic but requires substantial guidance to apply it. |
| 3 | Basic performance | Can perform the competency in familiar cases with some guidance. |

| | | |
|---|---|---|
| 4 | Competent in placement tasks | Can perform the competency in typical placement cases with minor guidance. |
| 5 | Confident and integrated performance | Can perform the competency independently and explain reasoning clearly in case discussion. |

## S2.3 Eleven-item self-rated core-competency questionnaire

| Item | Prompt | Scale | Relevance I-CVI | Clarity I-CVI |
|---|---|---|---|---|
| 1 | Identify key clinical information from a case. | 1-5 | 1.00 | 1.00 |
| 2 | Select appropriate first-round laboratory tests. | 1-5 | 1.00 | 1.00 |
| 3 | Explain the clinical significance of abnormal results. | 1-5 | 1.00 | 1.00 |
| 4 | Judge why laboratory results and clinical presentation do not match. | 1-5 | 1.00 | 1.00 |
| 5 | Propose supplementary or repeat testing. | 1-5 | 1.00 | 1.00 |
| 6 | Recognise pre-analytical, analytical, post-analytical or quality-control risks. | 1-5 | 1.00 | 1.00 |
| 7 | Translate laboratory findings into clinically useful judgement. | 1-5 | 1.00 | 1.00 |
| 8 | Feel confident contacting clinicians about suspicious results. | 1-5 | 1.00 | 1.00 |
| 9 | Explain reasoning clearly during group discussion. | 1-5 | 1.00 | 1.00 |
| 10 | Actively consult supporting information. | 1-5 | 1.00 | 1.00 |
| 11 | Revise reasoning after feedback. | 1-5 | 1.00 | 1.00 |

Total score range: 11-55; higher scores indicate stronger perceived competency. Historical end-of-placement item-level data showed Cronbach alpha = 0.92. The final verified intervention dataset retained pre- and post-course total scores rather than all intervention item-level responses, so intervention alpha was not recalculated in the revised manuscript.

## S2.4 Historical teaching-experience questionnaire

| Item | Prompt | Response / scoring |
|---|---|---|
| 1 | Learning objectives during rotations were clear. | Yes = 1, No = 0 |
| 2 | Teaching content was broadly consistent across sections. | Yes = 1, No = 0 |
| 3 | Traditional teaching helped link laboratory findings with clinical care. | Yes = 1, No = 0 |

| | | |
|---|---|---|
| 4 | Feedback was timely and specific. | Yes = 1, No = 0 |
| 5 | There were enough opportunities for case discussion. | Yes = 1, No = 0 |
| 6 | There were enough opportunities for direct communication with clinicians. | Yes = 1, No = 0 |
| 7 | Overall task load was acceptable. | Yes = 1, No = 0 |
| 8 | Would have chosen digital case-library teaching with dual mentors if available. | Yes = 1, No = 0 |

Support count range: 0-8. These data were used to describe perceived gaps in routine rotation-based teaching and were not used as a causal endpoint.

## S2.5 Intervention course-acceptability questionnaire

| Item | Prompt | Response / scoring |
|---|---|---|
| 1 | Mentor roles were clear. | Yes = 1, No = 0 |
| 2 | Cases were closely relevant to placement learning. | Yes = 1, No = 0 |
| 3 | Case difficulty was appropriate overall. | Yes = 1, No = 0 |
| 4 | Feedback was timely and specific. | Yes = 1, No = 0 |
| 5 | Teaching improved understanding of laboratory-clinical links. | Yes = 1, No = 0 |
| 6 | Teaching increased learning interest. | Yes = 1, No = 0 |
| 7 | Task load was acceptable. | Yes = 1, No = 0 |
| 8 | Would choose similar teaching again. | Yes = 1, No = 0 |

Acceptance count range: 0-8. These items were used to describe acceptability and learner engagement, not independent evidence of learning effectiveness.

## S2.6 Mentor rating form and anchors

| Score | Anchor for mentor-rated performance |
|---|---|
| 1 | Does not meet expected internship level; major errors or inability to explain reasoning. |
| 2 | Below expected level; task can be attempted only with substantial prompting. |
| 3 | Meets basic expected level; reasoning is understandable but incomplete. |
| 4 | Good performance; reasoning is generally accurate and communication is clear. |
| 5 | Excellent performance; integrated reasoning, quality-control awareness and communication are strong. |

| Retained rating domain | Operational definition | Score range |
|---|---|---|
| Overall performance | Integrated case reasoning and placement performance across the teaching programme. | 1-5 |
| Communication | Clarity, appropriateness and confidence of laboratory-clinician communication. | 1-5 |
| Quality-control reasoning | Recognition of pre-analytical, analytical, post-analytical and quality-control issues. | 1-5 |

Only the three aggregated mentor-rating variables shown above were retained in the final verified intervention dataset and analysed in the revised manuscript.

## S2.7 Final unseen-case objective assessment: candidate instructions and assessment material

Assessment format. Students completed a written, post-course, unseen case-based laboratory-clinical reasoning task. The case was not one of the 10 teaching cases. Students received a de-identified case stem and selected laboratory results and were asked to provide a written response. They were not told their mentor ratings, self-rating scores or rater identities. The assessment was used to evaluate case-linked reasoning after completion of the teaching programme.

| Candidate answer prompt | Maximum points |
|---|---|
| 1. Extract key clinical information and identify the main problem. | 10 |
| 2. State the main laboratory question or purpose of testing. | 10 |
| 3. Propose appropriate first-round or confirmatory laboratory tests. | 15 |
| 4. Interpret abnormal or discrepant results in relation to the clinical context. | 15 |
| 5. Judge whether the result is likely authentic or affected by pre-analytical, analytical, post-analytical or quality-control factors. | 15 |
| 6. Propose the next investigative step, including repeat testing, additional tests or specimen review when appropriate. | 10 |
| 7. Explain how the laboratory judgement would support clinical decision-making. | 15 |
| 8. Draft a concise communication note or verbal message to the clinician. | 10 |

De-identified final-assessment case-stem structure. The English text below reproduces the assessment structure and answer prompts in de-identified form. Dates, bed numbers, hospital numbers and any patient-identifiable details were removed. The task was designed to test transfer of reasoning to an unseen laboratory-clinical problem rather than recall of a teaching case.

| Case component | Material provided to students |
|---|---|
| Clinical context | A hospitalised adult has laboratory findings that appear inconsistent with part of the clinical picture. The clinician asks the laboratory whether the result can be used for immediate decision-making and what additional action is needed. |
| Laboratory data provided to students | A short table of selected haematology/chemistry/coagulation or relevant specialty results, including at least one abnormal or potentially discordant value, selected reference intervals and limited information on specimen condition or timing. |
| Student task | Students must identify key information, formulate the laboratory question, recommend first-round and follow-up tests, interpret the result, judge authenticity/quality-control risks, support clinical decision-making and communicate with the clinician. |
| Expected reasoning focus | Students should connect laboratory data with symptoms, timing, treatment context, specimen quality, analytical interference, repeat/confirmatory testing and communication of clinically useful judgement. |

## S2.8 Final unseen-case objective assessment rubric

| Domain | Max | Full-credit criteria | Partial-credit criteria | Low/zero-credit criteria |
|---|---|---|---|---|
| Information extraction / key clinical | 10 | Identifies all major clinical facts needed | Identifies some relevant facts but | Lists unrelated facts only or fails to extract |

| | | | | |
|---|---|---|---|---|
| information | | for interpretation, including symptoms, timing and relevant treatment or specimen context. | misses timing, treatment or specimen-context details. | clinically relevant information. |
| Understanding of the laboratory question / test purpose | 10 | Clearly states the core laboratory problem and what the clinician needs to know. | States the problem vaguely or incompletely. | Does not identify the laboratory question or misframes the problem. |
| First-round or confirmatory test selection | 15 | Selects appropriate tests and explains why they address the laboratory question. | Selects partially appropriate tests or gives limited rationale. | Selects inappropriate tests or no meaningful testing strategy. |
| Result interpretation | 15 | Interprets abnormal/discrepant results accurately in relation to clinical context and reference intervals. | Provides partly correct interpretation but lacks integration or misses key abnormality. | Misinterprets results or describes values without clinical meaning. |
| Authenticity / quality-control judgement | 15 | Systematically considers pre-analytical, analytical, post-analytical and quality-control explanations and distinguishes likely true versus false results. | Mentions some quality issues but reasoning is incomplete. | Does not consider quality-control or specimen/interference explanations. |
| Next investigative step | 10 | Provides a practical next step such as specimen review, repeat testing, confirmatory testing or additional clinically relevant tests. | Next step is possible but incomplete or poorly prioritised. | No appropriate next step. |
| Support for clinical decision-making | 15 | Explains how the laboratory judgement should influence clinical action or monitoring while acknowledging uncertainty. | Provides a general statement with limited decision relevance. | Does not connect the laboratory interpretation with clinical decision-making. |
| Laboratory-clinician communication | 10 | Provides a concise, professional message that explains the key result, uncertainty, recommended action and urgency. | Communication is understandable but incomplete or not prioritised. | Communication is unclear, inaccurate or absent. |

| Total score band | Interpretive anchor |
|---|---|
| 90-100 | Excellent integrated laboratory-clinical reasoning; accurate interpretation, strong quality-control awareness and clear clinician communication. |
| 80-89 | Good reasoning with minor omissions; interpretation and communication are mostly clinically useful. |
| 70-79 | Basic reasoning; important elements are present but integration, prioritisation or quality-control reasoning is incomplete. |
| 60-69 | Limited reasoning; major omissions in interpretation, next steps or communication. |
| <60 | Insufficient reasoning for safe independent laboratory-clinical judgement. |

## S2.9 Blinded scoring procedure

| Procedure element | Documentation |
|---|---|
| Anonymisation | Student names and identifiers were removed before scoring. Scripts were coded using anonymous study IDs. |
| Rater blinding | Raters were blinded to student identity, cohort/year, self-rated scores, mentor ratings and routine exit marks. |
| Scoring materials | Each rater received the candidate prompts, 100-point rubric and score-band anchors before scoring. |
| Calibration | No separate formal calibration dataset was retained in the final verified data. The written rubric and anchors were used to standardise scoring. |
| Final score | The final objective score was calculated directly as the mean of the two blinded raters total scores. |
| Domain-level data | The final verified dataset retained rater total scores and official mean total. Domain-level rater files were not retained; therefore, reliability is reported for total scores only. |

## S2.10 Content-validity documentation

| Element | Description |
|---|---|
| Expert panel composition | Seven anonymised reviewers examined the questionnaires and rubric: medical laboratory educators, clinical laboratory specialists, clinical mentors and educational-management staff. |
| Rating dimensions | Each item/domain was rated for relevance and clarity. |
| Rating rule | Experts used a 4-point relevance/clarity scale. Ratings of 3 or 4 were counted as content-valid. |
| I-CVI | Item-level content-validity index = number of experts rating an item as 3 or 4 divided by seven. |
| S-CVI/Ave | Scale-level average content-validity index = mean of item/domain I-CVI values. |
| Reported CVI values | The reported relevance and clarity CVI values of 1.00 refer to the final versions after wording standardisation and panel feedback. |

## S2.11 Open-ended feedback prompts

| Prompt | Response format | Analysis |
|---|---|---|
| What element of the programme was most helpful for your learning? | Free text | Two researchers independently coded de-identified response summaries into high-frequency themes. |
| What element of the programme most needs improvement? | Free text | Final themes were generated by combining similar codes and resolving discrepancies through discussion with a third author. |

Privacy note. Raw free-text comments, student identifiers and any patient-identifiable data are not included in the supplementary files. Additional documentation can be requested through the corresponding author subject to institutional educational data-governance approval and privacy protection.
